# Does professional identity play a critical role in the choice to remain in the nursing profession?

**DOI:** 10.1002/nop2.862

**Published:** 2021-03-14

**Authors:** Margareth Kristoffersen

**Affiliations:** ^1^ Department of Care and Ethics Faculty of Health Sciences University of Stavanger Stavanger Norway

**Keywords:** community of nurses, competence, professional identity, qualitative method, remaining in the nursing profession, written narratives

## Abstract

**Aim:**

To explore aspects of professional identity in nurses’ written narratives of what is significant to their choice to remain in the profession.

**Design:**

This study used a qualitative design and was underpinned by a hermeneutical approach.

**Methods:**

The participants were recruited via purposive sampling procedures and included 13 nurses aged 26 to 62 years. The data were collected in the form of written narratives to initiate the nurses’ reflections on the decision to remain in the profession. A thematic analysis was conducted.

**Results:**

The analysis revealed two themes: acting as a professional contributor and realigning to maintain professional belongingness. In the nurses’ written narratives, these aspects of professional identity were clearly significant to their choice to remain in the profession. In a complexly interwoven way, the aspects constructed who the nurses were as professionals, and thus, professional identity seems to play a critical role in the choice to remain.

## 1 INTRODUCTION

Research on the professional identity construction of nurses has indicated the relevance of professional identity to remaining in the nursing profession. Nonetheless, the link between professional identity and remaining in the profession needs to be more fully studied, and there seems to be little research on the aspects of professional identity that influence the choice to remain (Cho et al., 2010; Cowin & Hengstberger‐Sims, 2006; Cowin et al., 2008; Johnson et al., 2012), a desirable outcome in which nurses can achieve self‐realization, be engaged, be satisfied and have sense of belonging to an organization (Cho et al., 2010; Dwyer and Revell, 2016). The characteristics of the nurse, organization, colleagues, managers and work have influences (Brewer et al., 2012; Carter & Tourangeau, 2012; Cowden & Cummings, 2012; Jones et al., 2015), but the choice to remain in the nursing profession is obviously not a contingent matter. Instead, the choice seems to be based on nurses’ awareness of life choices and their ability to create benefits for both patient and themselves (Kristoffersen & Friberg, 2015, 2018). The growth and sustainability of the nursing profession depend on recruiting and retaining a new generation of professionals with a professional identity (Cowin & Hengstberger‐Sims, 2006; Horton et al., 2007; Johnson et al., 2012). Professional identity is vital to nurses, commences prior to nursing education and is constantly reshaped throughout nurses’ lifelong careers, implying that nurses reinforce and advance their identity (Johnson et al., 2012). In particular, nurse turnover, the process of nurses leaving the job and new nurses entering requires that nurses who remain in the profession have high engagement and excellent self‐efficacy (Moloney et al., 2018) and are trained to adequate professional skill levels (Yu et al., 2019). There has been a worldwide and dramatic increase in the demand for nurses (Hjemås et al., 2019; WHO, 2014; WHO, 2019). The World Health Organization recently stated that almost 6 million nurses are needed (WHO, 2020). In Norway, statistics have shown that there will be a shortage of 20,000 nurses by 2035 (Hjemås et al., 2019) and that one in five nurses leave their current positions in health services ten years after graduating with a bachelor's degree (Skjøstad et al., 2017).

## 2 BACKGROUND

Several theoretical frameworks have been used to understand the concept of professional identity (Briggs, 2007; Hunter et al., 2007; Trede et al., 2012). The concept is often linked to self‐concept (Hoeve and Roodbol, 2014; Johnson et al., 2012; Kroger & Marcia, 2011), which comprises cognitive and affective features, self‐awareness, self‐esteem, self‐worth and self‐confidence, or the way nurses think and feel about themselves. Thus, professional identity is perceived as an integral part of the personal identity considered a prerequisite for the process of professional identification (Öhlen & Segesten, 1998). The concept has been described as self‐identification with a profession (Gregg & Magilvy, 2001) or “the sense of being a professional” (Paterson et al., 2002, p.7), involving the common features of the professional discipline framework, professional knowledge discourses, values and standards of ethics (Cowin et al., 2013; Paterson et al., 2002; Kristoffersen et al., 2020). As the nursing profession is associated with traits such as caring, honesty and personal integrity (Bradby, 1990; Brennan & Timmins, 2012; Öhlen & Segesten, 1998), the professional identity of nurses has been defined as “what it means to be and act as a nurse; that is, it represents her/his philosophy of nursing,” and “the values and beliefs held by nurses that guide their thinking, actions and interactions with the patient” (Fagermoen, 1997, p.435). However, such features alone do not compose professional identity, and it has been stated that professional judgement, reasoning and critical self‐evaluation must be added (Paterson et al., 2002).

This study presents a view of professional identity not restricted to a self‐concept or self‐identification with the profession. It uses a social theory of learning perspective derived from the educational theorist Étienne Wenger (1998). In essence, Wenger perceives identity to be lived, meaning that identity is the core of human existence and is “fundamentally an experience that involves participation and reification” (1998, p.163). Wenger suggests that by bringing participation and reification together “through the negotiation of meaning, we construct who we are” (1998, p.151). Identity exists, then, “in the constant work of negotiating the self” (Wenger, 1998, p.151). Negotiability revolves around determining which meanings matter and involves the capacity “to shape the meanings that define our communities and our forms of belonging” (Wenger, 1998, p.145) or to make use of, control and modify “as ours the meaning that we negotiate,” to the degree that this is possible (Wenger, 1998, p.200).

Moreover, Wenger describes identity as “a pivot between the social and the individual” (1998, p. 145); in other words, identity is an intersection between the individual and the collective. It is nonetheless impossible to state exactly where the individual ends and the collective begins. The processes of identification become constitutive of an identity by “creating bonds or distinctions in which we become invested” (Wenger, 1998, p.191). Although such a process may not be directly addressed in narratives, Wenger (1998) claims that it resonates with narratives because narratives can transport experiences into the situations they relate to and can be integrated into one's identity. Put otherwise, there is a profound connection between narratives and identity. Narrative can be “integrated into our identities and remembered as personal experience, rather than as mere reification” (Wenger, 1998, p. 203–204). Developing a coherent narrative of one's life can be a way of shaping and reshaping professional identity and positioning oneself in a community with others.

Thus, the study contributes to discussions about how nurses’ professional identity, understood as lived, constituted and maintained by bonds between the individual and the collective, can play a critical role in the choice to remain in the nursing profession. The assumption was that identity might be reflected within the nurses’ written narratives. Their narratives may contain aspects of an identity formed by the enterprise of the nursing profession and articulated by the individual nurse even if professional identity is not directly addressed. The aim was to explore aspects of professional identity in nurses’ written narratives of what is important in their choice to remain in the nursing profession.

## 3 MATERIALS AND METHODS

### 3.1 Design

This study used a qualitative design (Polit & Beck, 2017) and was underpinned by a hermeneutical approach, with phenomenological openness as the interpretational attitude (Dahlberg et al., 2008). This perspective implies a shift from a natural attitude to a reflective attitude to avoid being misled by preconceptions and to remain open to data. The data were written narratives collected to elicit the participants’ reflection on the decision to remain in the nursing profession and to give them a point of departure in the subsequent data collection of a larger empirical study (Kristoffersen, 2013), involving the same participants but with two steps in the data collection and two separate data assessments. In the act of writing a narrative, reflection allows recorded events or meanings to be solidified and become clear (Dahlberg et al., 2008). The analysis involved movement from the whole to the parts and back again. The first interpretation was a tentative interpretation at a low level on the hermeneutic spiral, and the analysis moved up the hermeneutic spiral towards a more comprehensive understanding and, thus, the main findings (Dahlberg et al., 2008).

### 3.2 Participants

The participants were recruited via purposive sampling procedures (Polit & Beck, 2017). The following were eligible for inclusion: Registered Nurses with a minimum of two years of work experience, full‐time or almost full‐time work, within acute and long‐term physical and mental municipality and specialized health services, two participants from each of the different wards. Eligible participants were informed about the study by their formal leader. To ensure the participants’ willingness to talk about their experience, they were asked to contact the researcher themselves. The participants included 13 nurses aged 26 to 62 years. They had worked full time or almost full time for 2 to 40 years in municipality and specialized healthcare services (i.e., in nursing homes, home‐based care and mental, intensive, oncological, medical or surgical units). One to three participants were recruited from eight different wards. Many of the participants had worked for approximately ten years or more on the same ward. The participants included two men and 11 women. One participant graduated as a nurse, and twelve were postgraduates. Among the postgraduate participants, eight were educated as specialized nurses. Two nurses contacted the researcher without being included in the sample, one nurse was not eligible for inclusion, and one nurse contacted the researcher after the recruitment period and was therefore not included.

### 3.3 Data collection

The data were written narratives, defined as a personal account describing everyday experiences (Dahlberg et al., 2008). The main reason for this data collection in the larger empirical study (Kristoffersen, 2013) was to acquire a limited description of the participants’ experience of what was important in their choice to remain in the nursing profession and to help them focus on the study aim. The initial question the participants were asked was “If you were to tell me what is important for you to remain in nursing practice, what would you tell me?” The participants were asked to write as fully, concretely and precisely as possible and to send the narrative to the researcher via the Web.

## 4 ETHICAL CONSIDERATIONS

The study was approved by The Norwegian Centre for Research Data (no. 15577). Verbal and written information about the study was provided to the employed nurses considered eligible according to the inclusion criteria. Those who wished to participate in the study sent an e‐mail to the researcher. The researcher obtained written consent from the participants before participation. The participants were guaranteed confidentiality and notified about their right to withdraw. All the identifying information for the individual participants was removed.

### 4.1 Analysis

The data from 13 written narratives were one‐half to one‐and‐a‐half pages, single‐spaced, and were analysed across all the narratives. The analysis was based on the content of the text—“what” is said more than “how” it is said, the “told” rather than the “telling”—and on parts of the narrative that were thematically connected (Riesmann, 2008). Thus, the analysis took the form of reading and rereading the data to move beyond what the data said to what themes the nurses touched upon (Dahlberg et al., 2008). To arrive at a tentative understanding of the data, patterns of meaning were clustered, and meaning units that thematically seemed to belong to each other were consolidated. The next level of interpretation was to engage in a dialogue with the text and tentative interpretations to arrive at a more comprehensive or main understanding (Dahlberg et al., 2008). A main understanding was reached by comparing the tentative analyses considered valid to identify similarities and differences and recognize patterns that were previously or partly hidden. This part of the analysis required the use of a theoretical tool (Dahlberg et al., 2008), specifically that derived from Wenger (1998). By asking open questions of the text, such as “What do the participants tell about their daily experiences related to remaining in the nursing profession that can be understood as an expression of professional identity?”, “How do they build their professional identity, and what influences their identity regarding their remaining in the nursing profession?”, and “What does the process of identification involve while they remain in the profession?”, it was possible to go beyond the participants’ reflections.

## 5 FINDINGS

The analysis revealed the following aspects of professional identity contained in the narratives to be important in the nurses’ choice to remain in the profession: acting as a professional contributor and realigning to maintain professional belongingness.

### 5.1 Acting as a professional contributor

Professional contributions confirmed bonds to the community of nurses, and identity was constructed when the nurses could act as contributors among their colleagues. The relationship with colleagues provided the basis for participation. The nurses seem to feel “lucky” to work with skilled colleagues and achieve new skills:

For me, it is very important to achieve new skills and expand my work experience by contributing to colleagues’ further professional development through, for example, supervision.

Participation by sharing professional knowledge was considered salient, and it was described as important for the individual to transfer professional knowledge to colleagues. Making use of one's own professionality by sharing knowledge strengthened membership in the nursing community. The nurses could practice being an independent nurse and at the same time provide help to colleagues when needed. They had the autonomy to determine the content of their work and how it should be performed; however, as each patient should be treated as an individual, the nurses often discussed issues of relevance within the community when performing nursing care. Participation involved creating bonds among colleagues through a mutual exchange of skills and experience. The nurses identified colleagues’ investment in planning and coordinating patient care. It was appreciated that the nurses were willing to dedicate time, energy or other resources:

As nurses, we share skills and experience, and we help each other maintain professionality. We identify each other’s professional strengths and limitations and coordinate the tasks in line with the individual colleague’s resources.

Acting as a professional contributor provided intellectual stimulation. This role involves an invitation from colleagues to further develop one's professional knowledge so that all colleagues can work together. In particular, the student nurses who were enthusiastic newcomers who wanted to identify themselves as nurses provided intellectual stimulation. The student nurses provided what was described as “a breath of fresh air” when they posed questions and were curious, and thus, the nurses have to be intellectually sharp and aware of being a role model. A learning process was promoted whereby the nurses could act as contributors to the education of the student nurses by making use of their professional knowledge so that the students could construct a professional identity. Simultaneously, mentorship seemed to be one way the nurses maintained their own professional identity and involved finding meaning in the profession:

It is very inspiring to follow the student nurses, take part in their enthusiasm and how they develop professional knowledge, contribute to forming them and providing a base for their life as a nurse. It is important for me that they prefer to perform nursing care for the patient and find meaning in that work.

By providing a basis for the students’ professional enterprise related to maintaining and restoring the health of the patients, the nurses wanted to inspire the newcomers to stimulate their investment in the performance of nursing care and their bonds to the nursing profession. The nurses made it a priority to demonstrate for the student nurses how interesting the work can be within the field of nursing. For example, participants stated that it is enjoyable to work with older people.

### 5.2 Realigning to maintain professional belongingness

Belongingness was constructed through a process in which the nurses aligned themselves with their education and professional work. This aspect revolved around having a course of action achieved through education and advanced education:

Nursing is my education and my postgraduate education, and very early in my career, I found one field of nursing practice most interesting. I have worked within that field for 22 years. I still learn a lot and find my work informative and challenging.

A specific field of nursing practice constituted and maintained this nurse's course of action. Lifelong engagement in learning supported competence through continued development of professionality and facilitated the practice of nursing through the use of professional skills, that is, it provided the capacity to identify a patient's need for nursing care and take responsibility:

I’m competent as a nurse; I graduated as a nurse, and I have a postgraduate nursing degree and have worked as a nurse for 20 years. Often, I can see that what I’m doing as a professional nurse helps the patients and relieves their suffering, meaning that there is an outcome of what I’m doing.

Education and achieved professional knowledge complemented by professional updating gave this nurse a course of action. Staying professionally up to date was expressed as a bond to the enterprise of the nursing profession and provided a kind of power related to maintaining competence and continuing to be capable of implementing acceptable standards of nursing care. The following was stated:

For me, it is important to stay professionally up to date; that’s what the profession requires of me, and thus, to seek and learn new adequate knowledge to complement my previous experiences and skills.

Having a professional course of action was strongly emphasized and was linked to the ways the nurses thought and felt about themselves. Some nurses felt a natural caring for other human beings. They saw themselves as a social person and appreciated getting to know others. Notably, such a perspective can also be closely related to the choice to remain in the profession. Reflections that the profession fit with their natural strengths reflected a lifelong process of engagement in learning that was integrated into their professional identity:

I’m a kind of ‘learning’ human being and have a thirst for knowledge. During the years I have lived, I have obtained formal and informal professional knowledge, and I want to stay updated in my profession.

Realignment for belongingness depended on the nurses’ capacity to negotiate between what was expressed as “what is new and what is known.” Professional work will always contain familiar and understandable aspects of nursing care as well as new or more unknown aspects that require alignment if nurses are to remain. Basically, this process revolves around the sense of belonging. For the nurses, it was still amazing to learn new things, find new solutions and become more skilled, secure and safe to build upon their existing practices as a nurse. It was stated that the nursing profession builds on "a big portion" of the nurses’ creativity to never give the same nursing care to all the patients.

The nurses configured themselves as persons endowed with strength that was achieved through the continued performance of nursing care. This process involved vigorous personal growth within the nursing profession when they chose to remain in it. In planning, developing and monitoring nursing care, the patients’ desires, values and lifestyles were considered and managed through caring. The performance of holistic care based on person‐centredness incorporates a view of the patient as an equal partner:

As a professional nurse, I must be person‐centered and provide holistic nursing care, and this again has enriched and strengthened me as a person. I learn to know myself, my own reactions, thoughts and feelings. If I give much of myself, then I receive the same back.

Nevertheless, professional belongingness depended on recognition received from others. The nurses positioned themselves as professionals who need to be taken care of. They seemed to align their professional belongingness with being cared about and receiving recognition for their performance of nursing care: “I, as a nurse, need recognition for the nursing care I perform.” It was also stated explicitly that nurses and the nursing profession need to be taken care of:

We need to be taken care of as nurses. We need work conditions that promote safety and well‐being and reduce our stress. The nurses should be taken seriously. The patients need the nurses to be taken seriously. There are challenges within nursing practice that require attention, and if nurses are taken care of and remain in practice, the outcome can be job satisfaction, stability within the work force and qualified nursing care.

Professional belongingness included negotiating remaining in the nursing profession. Particularly when the nurses were tired, they questioned their belonging. However, the negotiations revealed that belongingness to the profession was precluded by other job opportunities. At the moment, in the nurses’ current position, other job opportunities could place a strain on them to adapt to another profession. This seems to be an expression of fact; other real, interesting job opportunities had not been offered. Such realigning involved pointing to the positive aspects of remaining in the profession. Working as a nurse was not necessarily “a dream job”; however, it was stated that many of the dreams related to what a good job should incorporate have come true.

## 6 DISCUSSION

The study illuminates how professional identity can play a critical role in the choice to remain in the nursing profession. Given that there is a connection between narratives and identity (Wenger, 1998), “acting as a professional contributor” and “realigning to maintain professional belongingness” were aspects of the nurses’ professional identity indicated in the written narratives to be important in the decision to remain in the profession. The study results support previous research asserting the relevance of professional identity to the choice to remain (Cowin & Hengstberger‐Sims, 2006; Horton et al., 2007; Johnson et al., 2012). However, the choice to remain is not seen as preferable based solely on reflections that the professional identity is consistent with one's self‐concept (Hoeve & Roodbol, 2014; Kroger & Marcia, 2011) or self‐identification with a profession (Gregg & Magilvy, 2001) or “the sense of being a professional” (Paterson et al., 2002, p.7). In the words of Wenger (1998), this study indicates how professional identity is lived. Wenger states that “an identity is neither narrowly local to activities nor abstractly global. Like practice, it is an interplay of both” (1998, p.163). The nurses constructed who they were as professionals, and individual nurses were positively influenced by the collective. Their identity involved negotiating ways of belonging to broader constellations and participating in broader discourses.

The theme “acting as a professional contributor” demonstrated how the studied nurses’ identity was constructed through the collective, that is relationships with nursing colleagues. Acting as a contributor strengthens bonds, which promotes being a member of a team and not an isolated professional decision maker. The studied nurses positioned themselves as highly competent. They defined who they were by participating in the nursing community; that is, they helped colleagues when needed, and they often discussed salient issues when performing nursing care. In Wenger's view, the participation was translated “into an identity as a form of competence” (1998, p.153). The nurses’ competence constituted accountability and contributed to shaping the meanings that matter within the nursing community. Wenger (1998) connects accountability to making an enterprise look at the world in certain ways and states that sustained engagement in a practice yields an ability to interpret and make use of the repertoire of that practice. This study supports previous research documenting the influence of collegial relationships, that is mentorship and teamwork, on nurses’ choice to remain (Ahlstedt et al., 2019; Karlsson et al., 2019; Van Osch et al., 2017). It also supports research documenting nurses’ impact on student nurses’ learning (Levett‐Jones et al., 2009) such that the students’ values and beliefs about the profession come to reflect those of the discipline (Johnson et al., 2012) and not the general public's image of nurses, which is often at odds with that of the nursing profession (Henderson, 1978; Hoeve & Roodbol, 2014; Öhlen & Segesten, 1998). Providing a basis for the student nurses’ practice and supporting the formation of their identity with respect to promoting, maintaining and restoring the health of patients also strengthened the nurses’ own professional identity. Thus, acting as a contributor in the community has a fundamental role in knowledge sharing and in stimulating colleagues’ further professional development that, in turn, influences the decision to remain in the profession.

The theme “realigning to maintain professional belongingness” also reflected how the studied nurses were influenced by the collective, that is how the nursing profession constituted and maintained their identity. The nurses had a professional course of action entailing earnest engagement and a lifelong process of continued learning based on a line of conduct, for example graduate and postgraduate degrees within the profession. The common enterprise of the profession seemed to be integrated into the studied nurses’ professional identity; that is, the professional discipline framework as described in ethical guidelines requires nurses to stay up to date in their profession (ICN, 2012). Staying professionally up to date has relevance to the professional knowledge discourses that emphasize actual applications of knowledge, such as technical and interpersonal skills requiring extensive training as well as professional judgement and critical self‐evaluation (Abbott, 1988; Briggs, 2007; Levett‐Jones et al., 2009; Paterson et al., 2002). Research has documented that nurses’ professional identity commences prior to nursing education and that the profession further forms nurses’ identity as they reinforce and advance their professional identity (Johnson et al., 2012). Nonetheless, the degree to which it was possible to belong depended on the individual nurses’ capacity to negotiate between new and already known professional knowledge. The modification of what was already known through the integration of the new with the already known advanced the studied nurses' understanding of who they were. In the view of Wenger (1998), negotiating involved the nurses realigning themselves to remain in familiar territory. Such realignment can be understood as an expression of how the nurses determined or obtained ownership of meaning: the nurses became able to control the meaning that mattered for their choice to remain. They aligned themselves with the traditional discourse of the nursing profession based on universal shared values and features such as care (Jones et al., 2015; Karlsson et al., 2019; Martinsen & Wyller, 2003; Öhlen & Segesten, 1998), with these values being “the foundation upon which the specific nature of identity generates” (Fagermoen, 1997, p.436). This implies that nurses’ professional identity relates to reification, that is the capacity, will and insight to integrate aspects of the profession's enterprise, such as person‐centeredness, into one's personal identity within the profession. A nurse with strong professionalism works in harmony with the enterprise of the profession and is aware of realizing professional knowledge, values and attitudes (Hwang et al., 2009; Jones et al., 2015).

Nevertheless, professional identity involves nurses’ negotiations related to remaining in the profession. Among the studied nurses, realigning to maintain belongingness involved positioning themselves as professionals with competence, and they wanted to be recognized for their provision of nursing care. The nurses did not feel the professional identity so strongly that they were independent of or no longer needed recognition for their competence. This finding supports that the choice to remain requires the realization of oneself as a nurse by creating something good for the patients and, moreover, for oneself (Kristoffersen, 2019; Kristoffersen & Friberg, 2015). However, nurses need permission from others and themselves to exercise self‐care and self‐compassion (Andrews et al., 2020). Providing nursing care and managing the emotions entailed in caring can be challenging, and if nurses are overwhelmed, burned out or unable to apply self‐care or self‐compassion, the risk is that they might be unable to provide high‐quality care for patients (Andrews et al., 2020). Such a risk can represent a threat to nurses’ professional identity, that is their self‐esteem, self‐worth and confidence (Hoeve and Roodbol, 2014; Johnson et al., 2012; Kroger & Marcia, 2011). This condition may heighten negotiations about remaining in the profession. The data demonstrate that the nurses did not necessarily consider the profession “a dream job,” but they had realized many work dreams, and consequently, adaptation to another profession would have been difficult. This finding suggests that remaining is concerned with an awareness of life choices. Understood as a life choice, remaining in the profession can be prioritized and evaluated as something more profound than not remaining (Kristoffersen & Friberg, 2018). A lifelong process of engagement was integrated into the nurses' professional identity.

### 6.1 Trustworthiness

The trustworthiness of the data is considered a strength, as the credibility of the data was ensured (Lincoln & Guba, 1985). The participants had several years of work experience. Their narratives recorded their reflections on paper, and while they were limited descriptions of their experience, they provided excellent examples of what was important for their choice to remain and thus reflected the basis of their professional identity. The narratives were written without interference from the researcher (Dahlberg et al., 2008). However, the participating nurses may have had a strong professional identity. A thematic approach was appropriate to identify the meaning units and what they revealed about the study aim. The analysis was conducted systematically, and the intended focus of the study was addressed. The framework of identity construction within communities of practice (Wenger, 1998) was a useful analytical tool. Openness was nonetheless practised in the analysis by remaining aware of the influence of the researcher's preconceptions (Dahlberg et al., 2008). Through the analysis, the study themes were confirmed (Lincoln & Guba, 1985). The themes were validated in the data, with the researcher verifying the relevance of the interpretations by lingering in uncertainty and avoiding premature conclusions based on the text. However, threats to confirmability arise when storied descriptions of experience do not reflect the meaning as experienced by participants (Polkinghorne, 2007). The complexity of experiences may not have been captured if the participants were reluctant to fully share their experiences due to the limitations of the written narratives. Furthermore, the research process has been sufficiently described, indicating that the data collection and analysis were conducted in a way that makes the study results dependable (Lincoln & Guba, 1985). The results are suggestive at best but may represent an overinterpretation of the limited data collected to study what was important in the nurses’ choice to remain in the profession. These features indicate that the results might not be transferable to other contexts, as transferability is “an empirical issue” (Lincoln & Guba, 1985, p.316). The study was reported based on the Consolidated Criteria for Reporting Qualitative Research (COREQ) guidelines (Tong et al., 2007).

### 6.2 Implications

Nurses’ remaining in the profession is critical for patients, as nurses improve care quality (Griffiths et al., 2018; Jones et al., 2015). Regarding the need to recruit and retain new generations of nurses (Hjemås et al., 2019; WHO, 2019; WHO, 2020) with a professional identity and expand the critical role of professional identity in the choice to remain (Cowin & Hengstberger‐Sims, 2006; Horton et al., 2007; Johnson et al., 2012), the nursing profession, and especially nursing leaders, have a pivotal role (Brewer et al., 2016; Carter & Tourangeau, 2012; Cowden & Cummings, 2012). The profession can acknowledge that professional identity does not rest solely on the individual nurse and that there seems to be a relationship between professional identity and remaining in the profession. Nursing leaders can promote a dialogue within the nursing community and with outsiders, for example organize a forum where they offer opportunities to collectively brainstorm approaches to developing professional identity by inviting nurses to thematize what is important to their choice to remain in the nursing profession and how to succeed in doing so. Framing in this way can provide a specific focus on how acting as a professional contributor in the nursing community reinforces bonds to the nursing profession and how belongingness to the enterprise of the profession can be realigned and maintained.

Additional research is also warranted. As nurses develop their identity over time, a longitudinal research design may be highly relevant for elucidating student nurses’ or newly educated nurses’ professional identity, especially regarding how the development of professional identity can influence the decision to remain.

## 7 CONCLUSION

The study highlights aspects of professional identity that seem to preclude leaving the nursing profession and thus indicate that professional identity plays a critical role in the choice to remain. Two aspects of professional identity were evident in the nurses’ written narratives: acting as a professional contributor in the nursing community and realigning to maintain professional belongingness. These aspects contributed in a complexly interwoven approach to constructing who the nurses were as professionals, implying that identity is lived and influences an outcome such as remaining in the nursing profession.

## CONFLICT OF INTEREST

I declare no conflicts of interest.

## AUTHOR CONTRIBUTION

Margareth Kristoffersen conceptualized the manuscript, collected the data, performed the analysis, drafted and revised the manuscript.

## Data Availability

The data that support the findings of this study are available from the corresponding author upon reasonable request.
